# IL-27 Signaling Is Crucial for Survival of Mice Infected with African Trypanosomes via Preventing Lethal Effects of CD4^+^ T Cells and IFN-γ

**DOI:** 10.1371/journal.ppat.1005065

**Published:** 2015-07-29

**Authors:** Gongguan Liu, Jinjun Xu, Hui Wu, Donglei Sun, Xiquan Zhang, Xiaoping Zhu, Stefan Magez, Meiqing Shi

**Affiliations:** 1 Division of Immunology, Virginia-Maryland Regional College of Veterinary Medicine, University of Maryland, College Park, Maryland, United States of America; 2 Department of Animal Genetics, Breeding and Reproduction, South China Agricultural University, Guangzhou, China; 3 Laboratory for Cellular and Molecular Immunology, Vrije Universiteit Brussels, Brussels, Belgium; 4 Structural Biology Research Centre, VIB, Brussels, Belgium; University of Manitoba, CANADA

## Abstract

African trypanosomes are extracellular protozoan parasites causing a chronic debilitating disease associated with a persistent inflammatory response. Maintaining the balance of the inflammatory response via downregulation of activation of M1-type myeloid cells was previously shown to be crucial to allow prolonged survival. Here we demonstrate that infection with African trypanosomes of IL-27 receptor-deficient (IL-27R^-/-^) mice results in severe liver immunopathology and dramatically reduced survival as compared to wild-type mice. This coincides with the development of an exacerbated Th1-mediated immune response with overactivation of CD4^+^ T cells and strongly enhanced production of inflammatory cytokines including IFN-γ. What is important is that IL-10 production was not impaired in infected IL-27R^-/-^ mice. Depletion of CD4^+^ T cells in infected IL-27R^-/-^ mice resulted in a dramatically reduced production of IFN-γ, preventing the early mortality of infected IL-27R^-/-^ mice. This was accompanied by a significantly reduced inflammatory response and a major amelioration of liver pathology. These results could be mimicked by treating IL-27R^-/-^ mice with a neutralizing anti-IFN-γ antibody. Thus, our data identify IL-27 signaling as a novel pathway to prevent early mortality via inhibiting hyperactivation of CD4^+^ Th1 cells and their excessive secretion of IFN-γ during infection with African trypanosomes. These data are the first to demonstrate the essential role of IL-27 signaling in regulating immune responses to extracellular protozoan infections.

## Author Summary

Infection with extracellular protozoan parasites, African trypanosomes, is characterized by a persistent inflammatory immune response. It has been recently shown that maintaining the balance of the inflammatory responses via dampening M1-type myeloid cell activation is critical to guarantee control of the parasites and survival of the host. In this study, we demonstrated that IL-27 receptor-deficient (IL-27R^-/-^) mice infected with African trypanosomes developed an excessive inflammatory response and severe liver immunopathology, resulting in dramatically reduced survival, as compared to infected wild-type mice. The early mortality of infected IL-27R^-/-^ mice was correlated with significantly elevated secretions of inflammatory cytokines, particularly IFN-γ, and enhanced activation of CD4^+^ Th1 cells. Importantly, IL-10 production was not impaired in infected IL-27R^-/-^ mice. Either depletion of CD4^+^ T cells, resulting in a dramatically reduced secretion of IFN-γ, or neutralization of IFN-γ, prevented the early mortality of infected IL-27R^-/-^ mice with a significantly reduced inflammatory response and a major amelioration of the liver pathology. Thus, our data identify IL-27 signaling as a novel pathway to prevent the early mortality via inhibiting hyperactivation of CD4^+^ Th1 cells and their excessive secretions of IFN-γ during experimental infection with extracellular protozoan parasites African trypanosomes.

## Introduction

African trypanosomiasis is a vector-borne parasitic disease of medical and veterinary importance. It is estimated that 170,000 people contract the disease every year, and that approximately 70 million people mainly in sub-Saharan Africa are at the risk of contracting the disease [1,2]. In addition, this disease severely limits the agricultural development by affecting domestic animals in the area [2]. The causative agents of this disease are various species of genus of *Trypanosoma*, which are extracellular protozoan parasites equipped with a flagellum that emerges from the flagellar pocket and provides the parasite with its motility [2]. Upon the bite of the mammalian host by a trypanosome-infected tsetse fly, the parasites enter the blood circulation via lymph vessels and can multiply in the bloodstream and interstitial fluids of the host [3,4]. The parasites have evolved very sophisticated evasion mechanisms to survive in the chronically infected host [3–5], causing a serious disease that is often fatal without treatment [1,2].

Due to practical and ethical reasons, mouse models have become an alternative and proven to be a cornerstone for studying African trypanosomiasis of humans and domestic animals [6]. Most of studies have been performed with *T*. *brucei* and *T*. *congolense* parasites [3,6]. Based on mouse models, although the parasites circulate in the blood stream, the liver is the major place for clearance of the parasites [7–9]. Recent studies demonstrated that Kupffer cells efficiently engulf trypanosomes, which is mediated by both IgM and IgG antibodies specific to the parasites [10–12]. IFN-γ, mainly secreted by VSG-specific CD4^+^ T cells [13–15] following activation by dendritic cells [16,17], has been shown to mediate protection during African trypanosomiasis [13,15,18–20]. Proinflammatory cytokines such as IL-12, TNF-α, as well as iNOS produced by M1-type myeloid cells are also critical for host resistance to African trypanosomes [15,21–25]. However, excessive secretions of these inflammatory cytokines by hyperactivated myeloid cells and T cells lead to liver pathology and shorten the survival of infected mice [11,22,26–29]. In this respect, IL-10 has been found to be essential for maintenance of the immunological balance between protective and pathological immune responses during African trypanosomiasis [11,20,22,26,27]. Importantly, the role of IL-10 as an anti-inflammatory agent has been more recently confirmed in cattle, primate and human infections with African trypanosomes [30–32]. It remains unknown whether, in addition to IL-10 signaling, another pathway that maintains this immunological balance exists.

IL-27, a recently identified cytokine produced primarily by macrophages and dendritic cells, is a member of the IL-12 super-family [33]. The IL-27 receptor (IL-27R) complex consists of the specific IL-27Rα subunit (WSX-1) and the IL-6R subunit (gp130), and is expressed on numerous subsets of leukocytes including CD4^+^ T cells, CD8^+^ T cells, NK cells, monocytes, Langerhans cells, and dendritic cells [34]. Earlier studies have demonstrated that IL-27, as a proinflammatory cytokine, drives naïve T cells to differentiate into Th1 cells [35–37]. More recent studies have suggested that IL-27 also has the function to inhibit immunopathology via downregulation of active CD4^+^ T cells during infections, particularly with intracellular protozoan parasites [38–42]. However, the precise mechanism of CD4^+^ T cell-mediated immunopathogenesis in the absence of IL-27 signaling still remains incompletely understood. In addition, it is not clear so far whether IL-27 plays an important role in regulation of the immune responses during infections with extracellular protozoan parasites such as African trypanosomes. Based on previous data showing that a subset of highly activated pathological CD4^+^ T cells produces excessive IFN-γ, and leads to immunopathology and early mortality of mice infected with *T*. *congolense* [11,28,29], we formulate a hypothesis that IL-27 signaling is, besides IL-10 signaling, another novel pathway that prevents the immunopathology and early mortality via down-regulation of the hyperactivity of CD4^+^ T cells and their excessive secretion of IFN-γ during experimental Africa trypanosomiasis. With this in mind, we examine in this study how IL-27 signaling regulates the immune responses in mice infected with African trypanosomes.

## Results

### 1. IL-27 signaling is crucial for survival of mice infected with *T*. *congolense*

**Fig 1:**
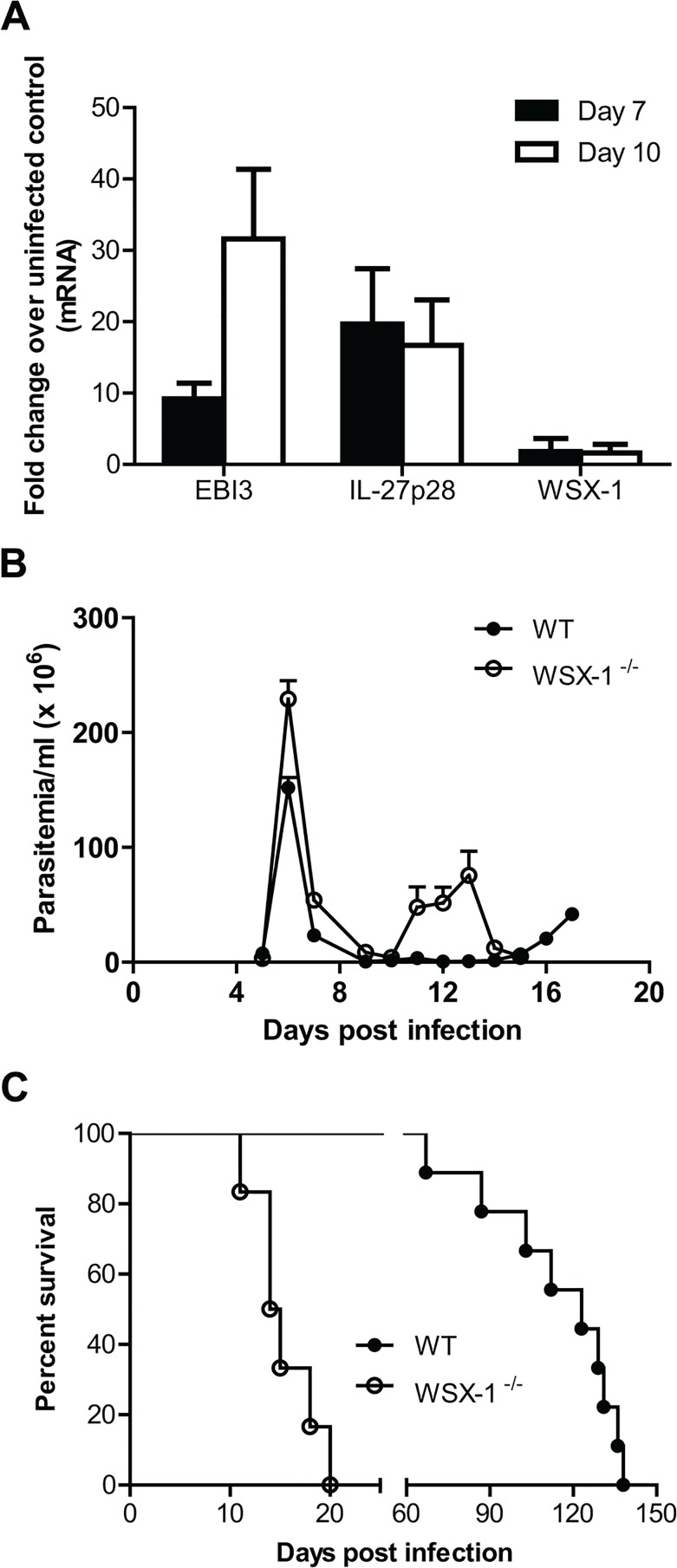
Enhanced expression of IL-27 and its crucial role in survival of mice infected with *T*. *congolense*. (A) mRNA expression levels of IL-27p28, EBI3 and WSX-1 in the liver of wild-type mice infected with *T*. *congolense* on day 7 and 10 versus day 0 (uninfected). (B) Parasitemia of IL-27R^-/-^ (WSX-1^-/-^) and wild-type mice (n = 6–9) infected with *T*. *congolense*. (C) Survival of IL-27R^-/-^ and wild-type mice (n = 6–9) infected with *T*. *congolense*. Data are presented as the mean ± SEM. The results presented are representative of 3 separate experiments.

To evaluate the role of IL-27 signaling during African trypanosomiasis, we first determined whether infection led to increased expression of this cytokine or its receptor. Wild-type C57BL/6 mice were infected with *T*. *congolense*, a species of African trypanosomes which are unable to leave the circulation and only live in blood vessels, causing fatal disease in cattle [4]. The mice were euthanized at day 0, 7, and 10 after infection, as parasitemia usually peaked on day 6–7 after infection [15,29]. As the liver is the major organ for clearance of the parasites [7–9,11], the liver was collected for measurement of mRNA levels of IL-27 and its receptor using real-time quantitative RT-PCR. mRNA levels of both subunits of IL-27 (IL-27p28 and EBI3) were upregulated in the liver of mice at day 7 and 10 after infection, compared to uninfected mice (Fig 1A). In contrast, mRNA levels of IL-27 receptor (WSX-1) were not affected by the infection (Fig 1A).

Next, we infected IL-27R^-/-^ (WSX-1^-/-^) and wild-type mice with *T*. *congolense* to assess whether IL-27 signaling affected the disease progression. Similar to infected wild-type mice, infected IL-27R^-/-^ mice could control the first wave of parasitemia (Fig 1B). However, IL-27R^-/-^ mice succumbed to the infection on day 12 to 20 after infection with a mean survival time of 14.5 days (Fig 1C). In contrast, infected wild-type mice survived until day 67 to 138 days after infection with a mean survival time of 123 days (Fig 1C). Compared to infected wild-type mice, the infected IL-27R^-/-^ mice survived significantly shorter (p<0.01). These data demonstrated that IL-27 signaling is required for survival of mice infected with *T*. *congolense*.

### 2. Deficiency of IL-27 signaling results in enhanced systemic inflammatory responses in mice infected with *T*. *congolense*

**Fig 2:**
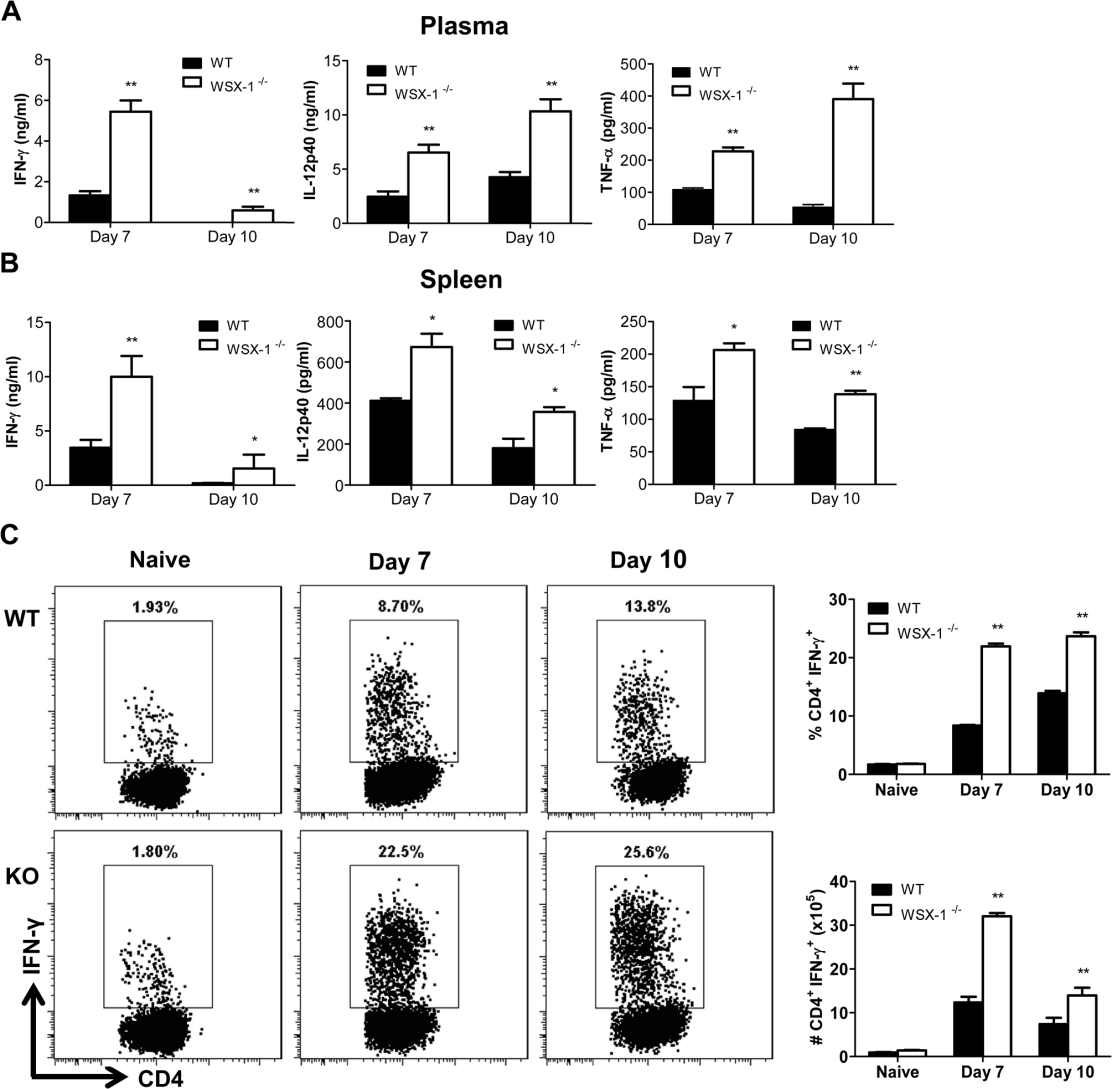
IL-27 signaling suppresses systemic inflammatory responses in mice infected with *T*. *congolense*. (A) Plasma levels of IFN-γ, IL-12p40, and TNF-α in IL-27R^-/-^ (WSX-1^-/-^) and wild-type mice (n = 4) on day 7 and 10 after infection with *T*. *congolense*. (B) Secretions of IFN-γ, IL-12p40 and TNF-α in the supernatant fluids of cultured spleen cells purified from IL-27R^-/-^ and wild-type mice (n = 4) on day 7 and 10 following infection with *T*. *congolense*. (C) The frequency (left and upper right) and the absolute number (lower right) of splenic IFN-γ-producing CD4^+^ T cells derived from IL-27R^-/-^ and wild-type mice (n = 3) on day 0, 7 and 10 after infection following 12 h *in vitro* restimulation with Cell Stimulation Cocktail (containing PMA, ionomycin, and protein transport inhibitors). Data are presented as the mean ± SEM. The results presented are representative of 2–3 separate experiments.

The above results demonstrated that absence of IL-27 signaling led to earlier mortality of mice infected with African trypanosomes. As uncontrolled inflammation causes early mortality of mice infected with African trypanosomes [3,4], we next examined the plasma levels of inflammatory cytokines and their secretions by cultured spleen cells. As shown in Fig 2A, significantly higher amounts of IFN-γ, IL-12p40, and TNF-α were detected in the plasma of IL-27R^-/-^ mice infected with *T*. *congolense*, compared to infected wild-type mice, on day 7 and 10 after infection (p<0.01). Although the plasma level of IFN-γ in IL-27R^-/-^ mice decreased on day 10 after infection probably due to clearance of the first wave of parasitemia, it was still significantly higher than that of the infected wild-type mice (p<0.01, Fig 2A).

To evaluate the secretions of cytokines by spleen cells, spleen cells were collected from IL-27R^-/-^ and wild-type mice on day 7 and 10 after infection with *T*. *congolense*, and cultured *in vitro* for 48 h. The production of IFN-γ, IL-12p40, and TNF-α by spleen cells were significantly elevated in infected IL-27R^-/-^ mice, compared to infected wild-type mice (p<0.01 or <0.05, Fig 2B). As recent studies have shown that IL-27 mainly regulates CD4^+^ T cell activation during infection with intracellular pathogens [38–42], we further evaluated IFN-γ-producing CD4^+^ T cells in the spleen cultures using flow cytometry. A limited and similar percentage and absolute number of CD4^+^ T cells from uninfected wild-type and IL-27R^-/-^ mice produced IFN-γ after 12 h stimulation with Cell Stimulation Cocktail (containing PMA, ionomycin, and protein transport inhibitors). However, by 7 and 10 days post infection both the percentage and the absolute number of IFN-producing CD4^+^ T cells were significantly enhanced in IL-27R^-/-^ mice when compared to wild-type cohorts (Fig 2C).

### 3. IL-27R^-/-^ mice develop severe liver pathology during infection with *T*. *congolense*

**Fig 3:**
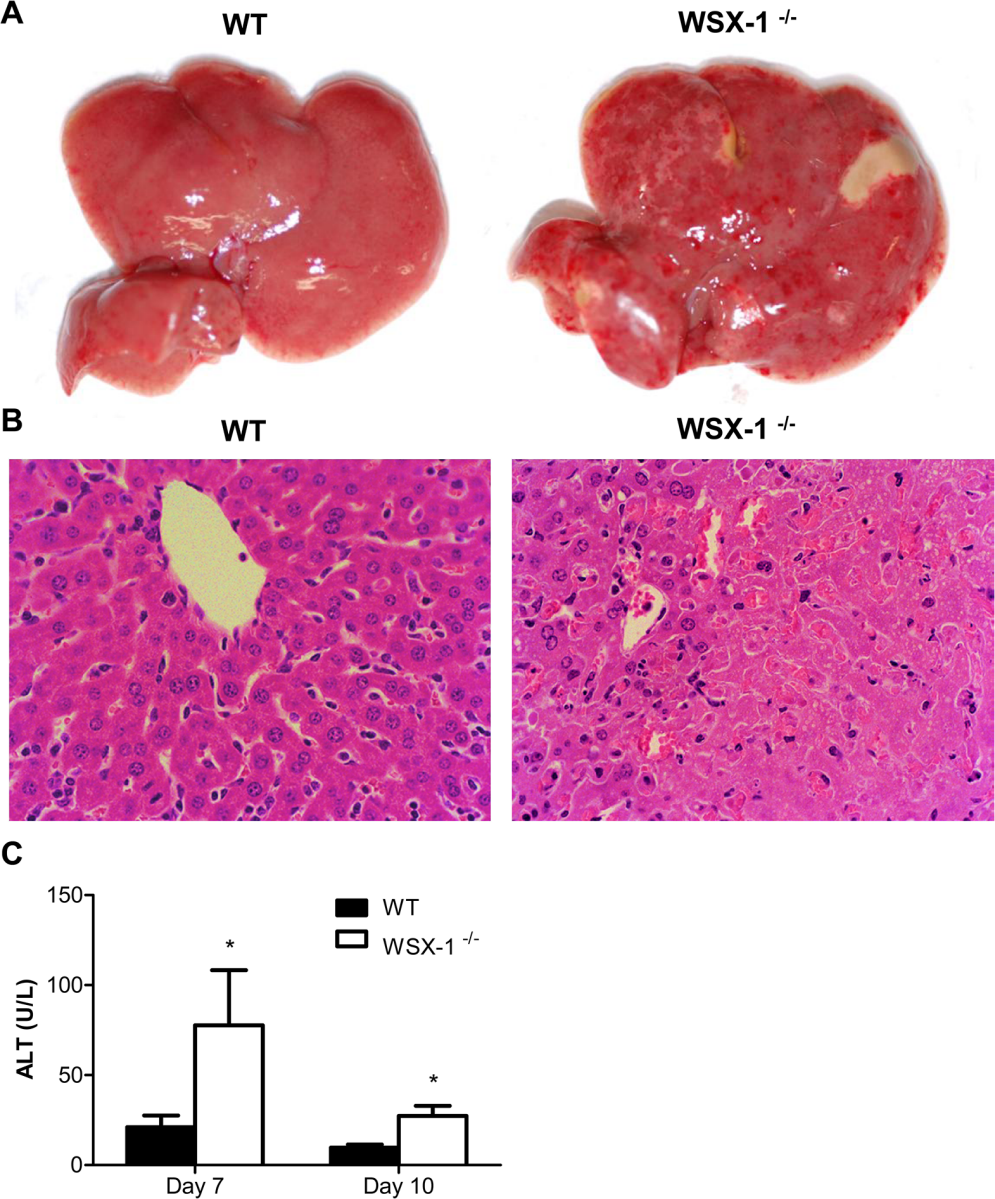
IL-27 signaling is required to prevent liver immunopathology during infection with *T*. *congolense*. (A) Macroscopic examination of liver on day 10 after infection with *T*. *congolense* revealed the presence of extensive pale geographic areas in IL-27R^-/-^ (WSX-1^-/-^), but not wild-type mice (n = 4). (B) Hematoxylin and eosin staining showing loss of hepatocyte cellular architecture in the liver of IL-27R^-/-^, but not wild-type mice (n = 4) on day 10 after infection with *T*. *congolense* (original magnification ×40). (C) Serum ALT activities were assessed in IL-27R^-/-^ and wild-type mice (n = 4) on day 7 and 10 after infection with *T*. *congolense*. Data are presented as the mean ± SEM. The results presented are representative of 2 separate experiments.

We and others have previously shown that excessive systemic inflammatory responses of mice infected with African trypanosomes are associated with severe liver damage [11,22,43,44]. In addition, the liver is the primary organ of trypanosome clearance [7,9,11]. Therefore, we next evaluated effects of IL-27 signaling on liver pathology during the course of infection with the parasites. IL-27R^-/-^ mice, but not wild-type mice, showed extensive pale geographic areas highly suggestive of necrosis on day 10 after infection with *T*. *congolense* (Fig 3A). Microscopic examination of the liver of infected IL-27R^-/-^ mice revealed many large areas with loss of hepatocyte cellular architecture and an infiltration of inflammatory cells (Fig 3B). By contrast, these pathological changes were not observed in the liver of infected wild-type mice (Fig 3B). To further characterize the liver pathology, we measured the serum activities of alanine aminotransferase (ALT) of mice during *T*. *congolense* infection. As shown in Fig 3C, IL-27R^-/-^ mice had significantly higher serum activities of ALT than wild-type mice on both day 7 and day 10 after infection (p<0.05), indicating death of hepatocytes and release of cytosolic enzymes. These results demonstrated that IL-27 signaling played a major role in prevention of the liver pathology that was associated with enhanced systemic inflammatory responses.

### 4. Early mortality of IL-27R^-/-^ mice infected with *T*. *congolense* is not due to impaired IL-10 production

**Fig 4:**
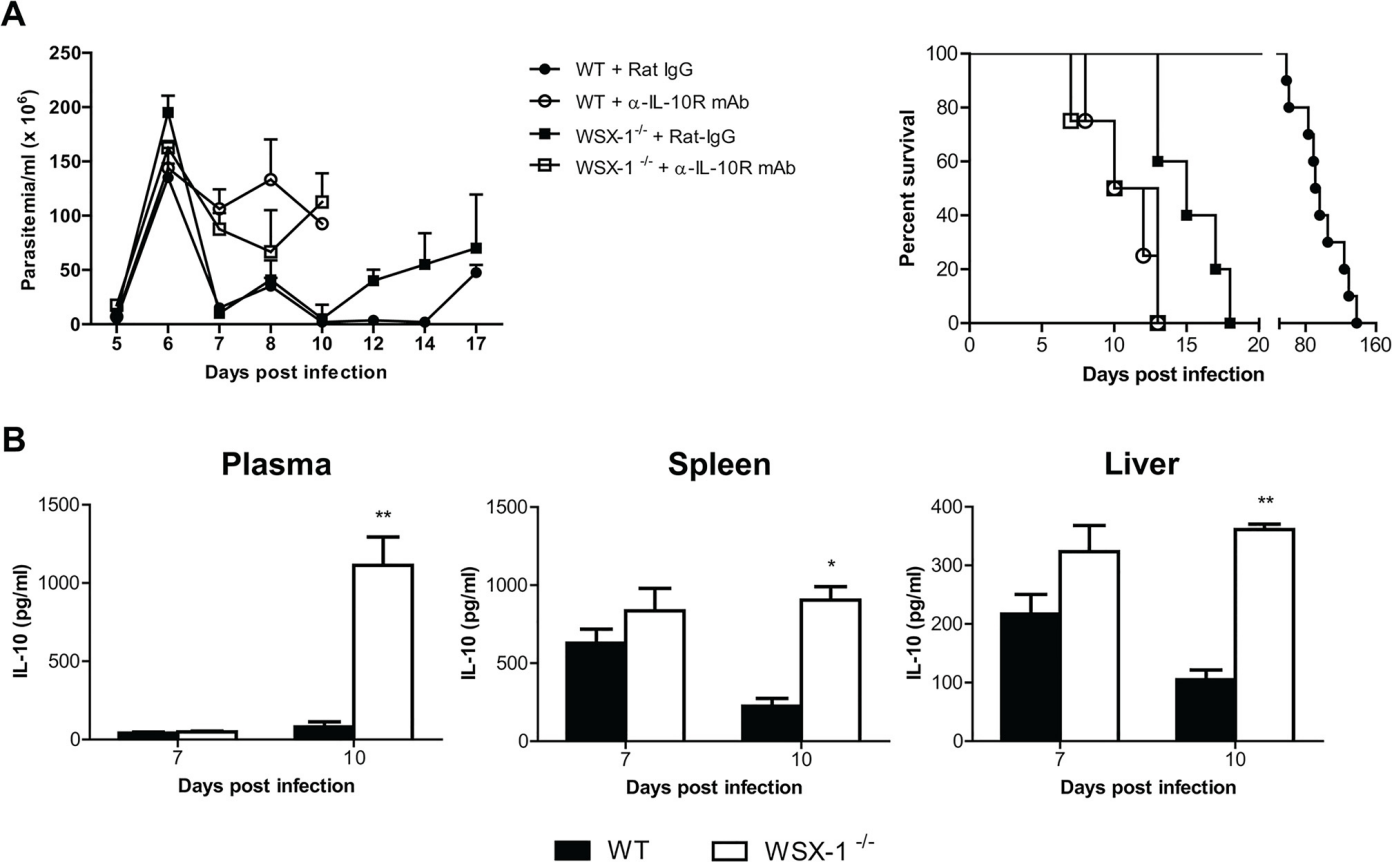
IL-10 production is not impaired in IL-27R^-/-^ (WSX-1^-/-^) mice infected with *T*. *congolense*. (A) Parasitemia and survival of IL-27R^-/-^ and wild-type mice (n = 4–10) treated with 0.4 mg anti-IL-10R mAb or rat IgG on day 0, 2, 4, and 6 after infection with *T*. *congolense*, respectively. (B) IL-10 levels in plasma, and supernatant fluids of cultured spleen cells, and liver leukocytes from IL-27R^-/-^ and wild-type mice (n = 4) on day 7 and 10 after infection with *T*. *congolense*. Data are presented as the mean ± SEM. The results presented are representative of 2–3 separate experiments.

It has been shown that IL-10 is crucial for survival of mice infected with African trypanosomes through limiting inflammation [11,20]. In particular, failure to control inflammatory responses in mice infected with African trypanosomes in the absence of IL-10 signaling is associated with severe liver pathology [11,22,27]. In this regard, IL-27 has been shown to drive CD4^+^ T cells to produce IL-10 for downregulation of inflammation [45–47]. The similarity of the cytokine profile and liver pathology of infected mice in the absence of IL-27 signaling and IL-10 signaling [11,20] prompted us to examine whether IL-27 signaling prevented early mortality of mice infected with African trypanosomes via IL-10. We first compared the disease progression in the absence of IL-27 signaling with that in the absence of IL-10 signaling. *T*. *congolense*-infected IL-27R^-/-^ mice and wild-type mice showed similar parasitemia and a significantly reduced survival after administration of anti-IL-10 receptor (IL-10R) mAb (p<0.01, Fig 4A). Strikingly, infected wild-type mice treated with anti-IL-10R mAb survived significantly shorter than infected IL-27R^-/-^ mice (p<0.01, Fig 4A), suggesting that IL-27 and IL-10 may independently regulate inflammatory responses during African trypanosomiasis. Next we compared the IL-10 levels in plasma, and supernatant fluids of cultured spleen cells or liver leukocytes between IL-27R^-/-^ and wild-type mice infected with *T*. *congolense*. There was no significant difference in IL-10 production in plasma and supernatant fluids of the cultures between IL-27R^-/-^ and wild-type mice on day 7 after infection (Fig 4B). Surprisingly, IL-27R^-/-^ mice even showed significantly higher amounts of IL-10 in both plasma (up to 14 folds) and supernatant fluids of cultured spleen cells or liver leukocytes on day 10 after infection (p<0.01 or <0.05, Fig 4B), demonstrating that secretion of IL-10 was strengthened, rather than impaired in IL-27R^-/-^ mice infected with African trypanosomes, probably due to deficiency of the immune regulation mediated by IL-27 signaling in those infected IL-27R^-/-^ mice. Taken together, these data suggested that early mortality of IL-27R^-/-^ mice infected with African trypanosomes was not due to impaired IL-10 production.

### 5. Enhanced CD4^+^ T cell responses and elevated secretions of inflammatory cytokines in the liver of IL-27R^-/-^ mice infected with *T*. *congolense*

**Fig 5:**
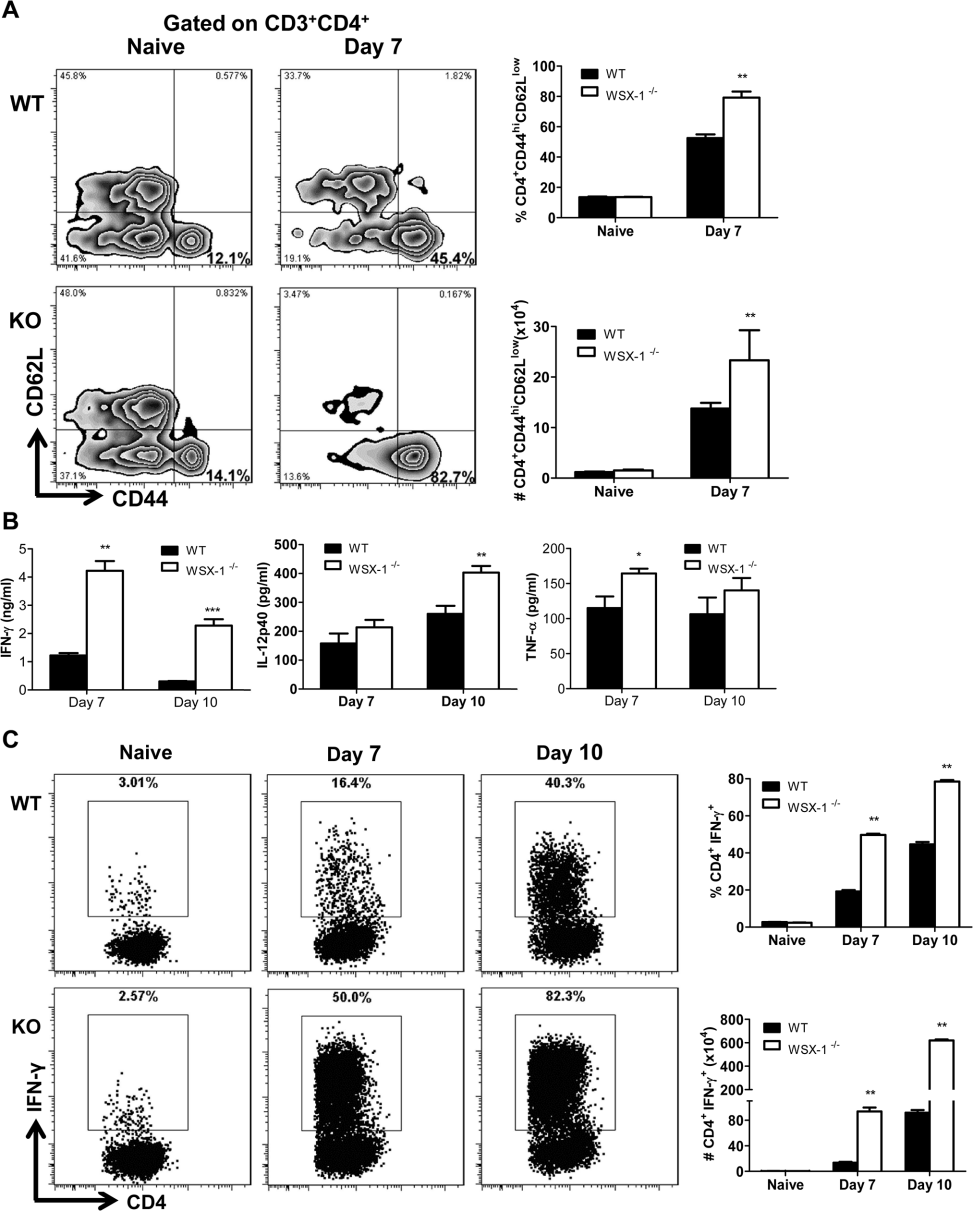
Enhanced activation of CD4^+^ T cells and elevated production of inflammatory cytokines in the liver of IL-27R^-/-^ (WSX-1^-/-^) mice infected with *T*. *congolense*. (A) The frequency (left and upper right) and the absolute number (lower right) of activated CD4^+^ T cells (CD44^hi^CD62L^low^) derived from the liver of IL-27R^-/-^ and wild-type mice (n = 3) on day 0 and 7 after infection with *T*. *congolense*. (B) Production of IFN-γ, IL-12p40, and TNF-α in the supernatant fluids of cultured liver leukocytes purified from IL-27R^-/-^ and wild-type mice (n = 4) on day 7 and 10 after infection with *T*. *congolense*. (C) The frequency (left and upper right) and the absolute number (lower right) of IFN-γ-producing CD4^+^ T cells derived from the liver of IL-27R^-/-^ and wild-type mice (n = 3) on day 0, 7 and 10 after infection with *T*. *congolense* following 12 h *in vitro* restimulation with Cell Stimulation Cocktail (containing PMA, ionomycin, and protein transport inhibitors). Data are presented as the mean ± SEM. The results presented are representative of 2–3 separate experiments.

Because early mortality of IL-27R^-/-^ mice infected with African trypanosomes was associated with severe liver pathology without impaired secretion of IL-10 as shown above and because IL-27 has been shown to mainly regulate T cell, particularly CD4^+^ T cell activation during infection with intracellular pathogens [38–42], we next characterized CD4^+^ T cell responses in the liver of IL-27R^-/-^ mice during infection with *T*. *congolense*. We found that the frequency and the absolute number of activated hepatic CD4^+^ T cells (CD44^hi^CD62L^low^) were significantly higher in IL-27R^-/-^ mice infected with *T*. *congolense*, compared to infected wild-type mice (p<0.01, Fig 5A). The production of IFN-γ, IL-12p40, and TNF-α by cultured liver leukocytes from infected IL-27R^-/-^ mice was significantly higher than production of these cytokines by liver leukocytes from infected wild-type mice (p<0.001, <0.01 or <0.05, Fig 5B). In particular, the production of IFN-γ was enhanced by 4–8 folds in the liver leukocyte cultures of infected IL-27R^-/-^ mice (Fig 5B). Thus, we further evaluated the activation of liver CD4^+^ T cells by examining their secretions of IFN-γ using single cell analysis. A small and similar percentage and absolute number of CD4^+^ T cells from uninfected wild-type and IL-27R^-/-^ mice secreted IFN-γ after 12 h stimulation with Cell Stimulation Cocktail (containing PMA, ionomycin, and protein transport inhibitors). In contrast, by day 7 and 10 post infection significantly higher percentage and absolute number of IFN-γ-producing CD4^+^ T cells were detected in IL-27R^-/-^ mice as compared to wild-type cohorts (Fig 5C). Collectively, these data suggested that the early mortality of IL-27R^-/-^ mice infected with African trypanosomes was associated with exacerbated Th1-mediated immune responses with overactivation of CD4^+^ T cells.

### 6. CD4^+^, but not CD8^+^, T cells mediate the early mortality of IL-27R^-/-^ mice infected with *T*. *congolense*

**Fig 6:**
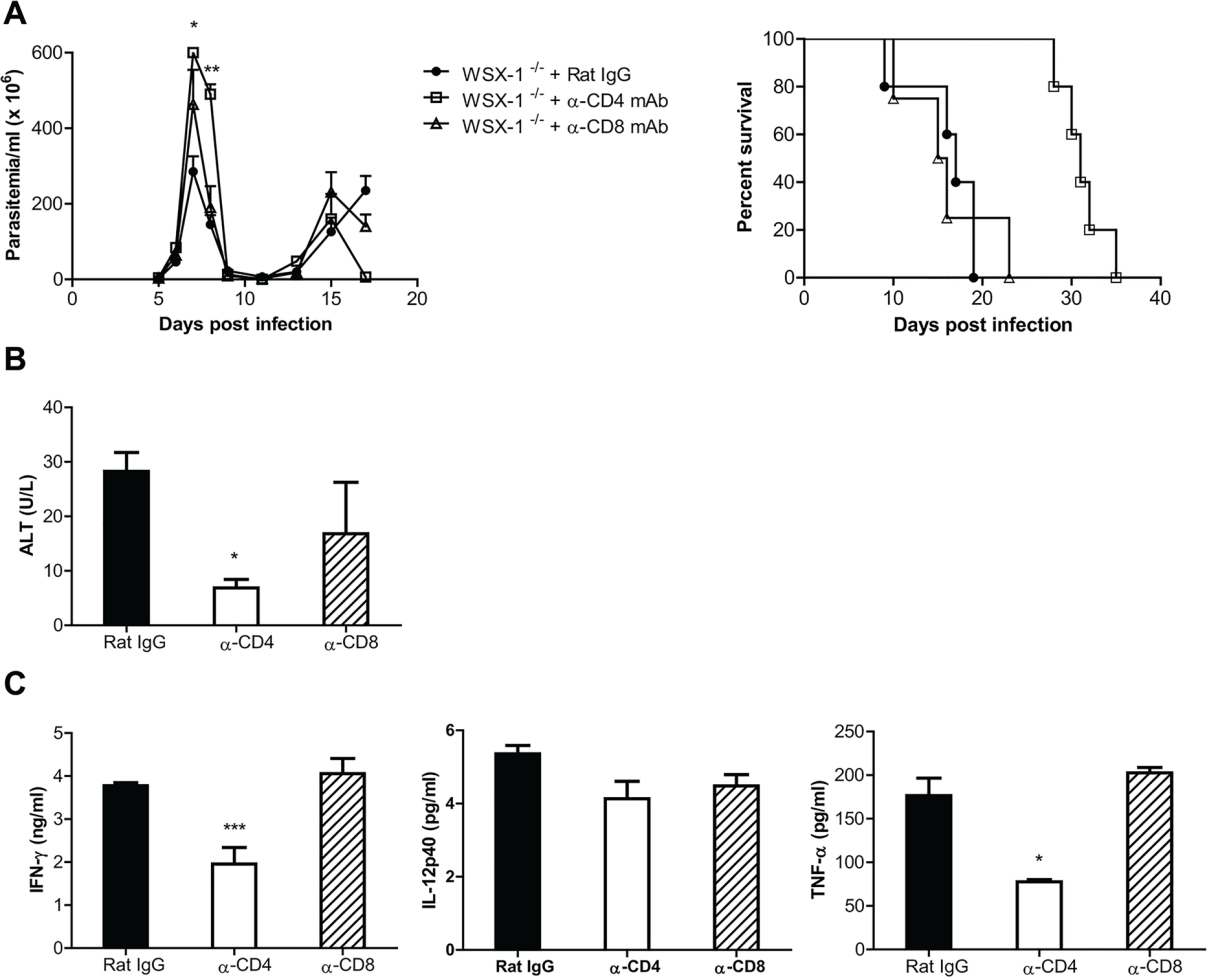
Depletion of CD4^+^, but not CD8^+^, T cells significantly reduces the production of inflammatory cytokines and the serum activities of ALT, and enhances the survival of IL-27R^-/-^ (WSX-1^-/-^) mice infected with *T*. *congolense*. IL-27R^-/-^ mice were infected with *T*. *congolense*, and treated with 0.5 mg rat anti-mouse CD4 mAb, rat anti-mouse CD8 mAb, or rat IgG on day 0, 2, 4, and 6 after infection, respectively. (A) Parasitemia and survival of the IL-27R^-/-^ mice (n = 4–5) infected with *T*. *congolense*. (B) Serum ALT activities were assessed in IL-27R^-/-^ mice (n = 4) on day 7 after infection with *T*. *congolense*. (C) Plasma levels of IFN-γ, IL-12p40, and TNF-α of IL-27R^-/-^ mice (n = 4) on day 7 after infection with *T*. *congolense*. Data are presented as the mean ± SEM. The results presented are representative of 2 separate experiments.

As shown above, CD4^+^ T cells were excessively activated in the liver of IL-27R^-/-^ mice infected with African trypanosomes, raising the possibility that the early mortality of infected IL-27R^-/-^ mice was a consequence of a CD4^+^ T cell-dependent immune-mediated pathology. To test this, IL-27R^-/-^ mice infected with *T*. *congolense* were treated with depleting anti-mouse CD4 mAb, anti-mouse CD8 mAb, or rat IgG as control; and the course of infection, immune responses, and severity of liver damage were assessed. As shown in S1 Fig, administration of the antibodies efficiently depleted CD4^+^ T cells or CD8^+^ T cells in the spleen and liver of the infected mice. Infected mice from all three groups could effectively control the first wave of parasitemia, although depletion of CD4^+^ T cells resulted in a significantly higher parasitemia at some time points of infection (p<0.01 or <0.05, Fig 6A). Strikingly, infected IL-27R^-/-^ mice treated with anti-CD4 mAb had two fold increase of survival compared to infected IL-27R^-/-^ mice treated with rat IgG (p<0.01, Fig 6A). In contrast, depletion of CD8^+^ T cells did not affect the survival of infected IL-27R^-/-^ mice (Fig 6A). These results demonstrated that IL-27 signaling had a crucial role in dampening CD4^+^ T cell activation in experimental *T*. *congolense* infection in mice, allowing for prolonged survival.

We next evaluated the effect of CD4^+^ T cells on weight loss and liver pathology of IL-27R^-/-^ mice infected with *T*. *congolense*. Infected IL-27R^-/-^ mice treated with anti-CD4 mAb had significantly less weight loss at the later stage of infection, compared to infected IL-27R^-/-^ mice treated with rat IgG or anti-CD8 mAb (p<0.01; S2A Fig). Importantly, infected IL-27R^-/-^ mice treated with rat IgG or anti-CD8 mAb exhibited many large areas with loss of hepatocyte cellular architecture in the liver, whereas these pathological changes were hardly seen in the liver of infected IL-27R^-/-^ mice treated with anti-CD4 mAb (S2B Fig). In addition, depletion of CD4^+^, but not CD8^+^, T cells significantly reduced the serum activities of ALT in IL-27R^-/-^ mice infected with *T*. *congolense* (p<0.05, Fig 6B). These data suggested that CD4^+^ T cells played a central role in the development of liver pathology in experimental *T*. *congolense* infection, and that IL-27 was crucial for dampening this CD4^+^ T cell-mediated pathology.

We further characterized the contributions of CD4^+^ T cells to secretion of cytokines in IL-27R^-/-^ mice infected with *T*. *congolense*. Depletion of CD4^+^, but not CD8^+^, T cells significantly reduced plasma levels of IFN-γ and TNF-α in infected IL-27R^-/-^ mice (p<0.001 or <0.05), although the reduction of IL-12p40 did not reach statistical significance (Fig 6C). In addition, depletion of CD4^+^, but not CD8^+^, T cells also resulted in significantly less secretion of IFN-γ by spleen cells from infected IL-27R^-/-^ mice (p<0.05, S2C Fig). Interestingly, depletion of CD4^+^ T cells almost abrogated the production of IL-10 by spleen cells in infected IL-27R^-/-^ mice (p<0.01, S2C Fig), suggesting that IL-10 was predominantly produced by CD4^+^ T cells. Importantly, the observation that the enhanced survival of infected IL-27R^-/-^ mice treated with anti-CD4 mAb was correlated with very little secretion of IL-10 further suggested that IL-27 signaling inhibited hyperactivation of Th1 cells in an IL-10 independent manner as shown above in Fig 4.

### 7. Neutralization of IFN-γ prevents the early mortality of IL-27R^-/-^ mice infected with *T*. *congolense*

**Fig 7:**
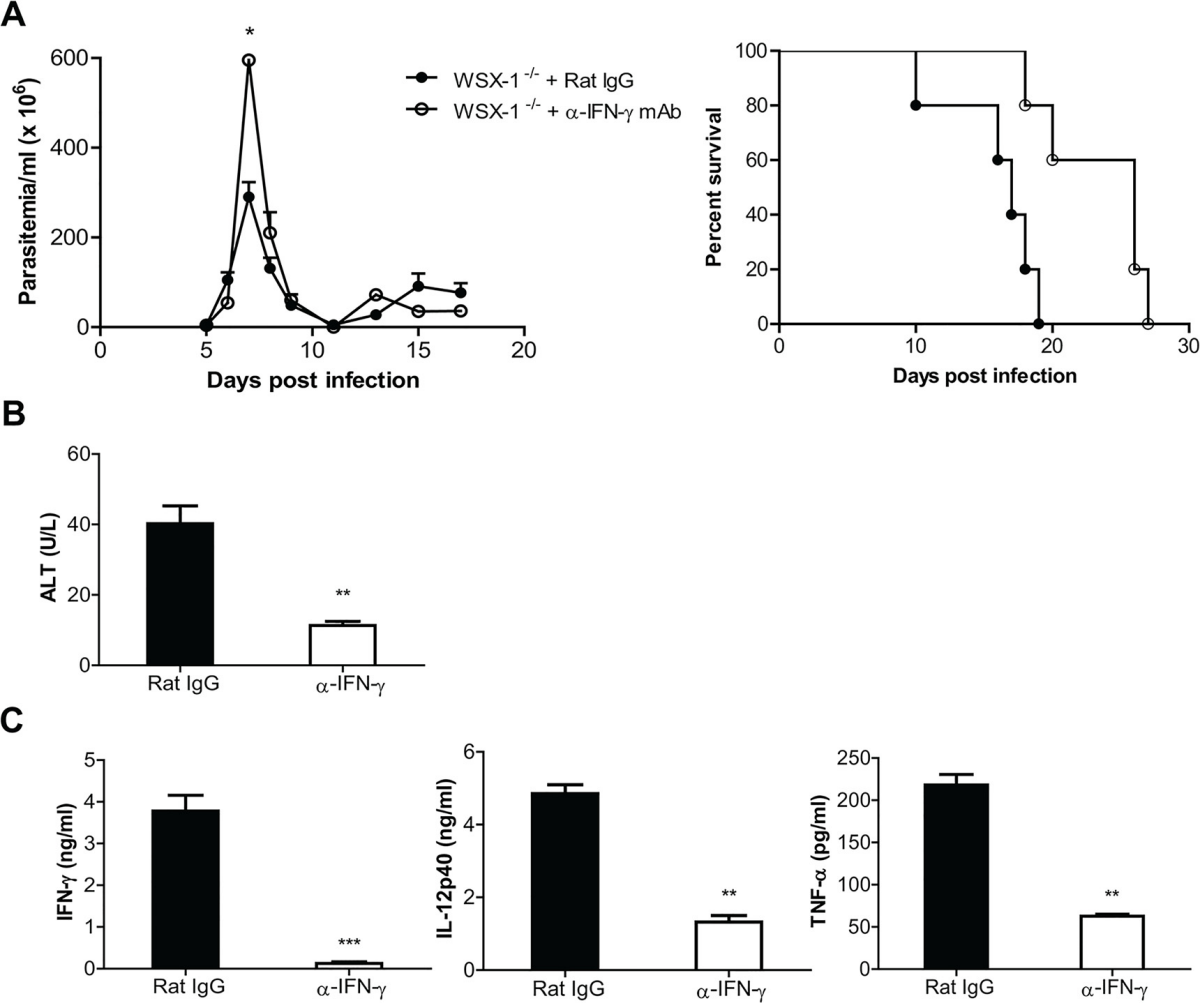
Neutralization of IFN-γ significantly reduces the production of inflammatory cytokines and the serum activities of ALT, and prevents the early mortality of IL-27R^-/-^ (WSX-1^-/-^) mice infected with *T*. *congolense*. IL-27R^-/-^ mice were infected with *T*. *congolense*, and treated with 0.4 mg rat anti-mouse IFN-γ mAb or rat IgG on day 0, 2, 4, 6, 8, 10, 12, and 14 after infection, respectively. (A) Parasitemia and survival of infected IL-27R^-/-^ mice (n = 5). (B) Serum ALT activities were assessed in IL-27R^-/-^ mice (n = 4) on day 7 after infection. (C) Plasma levels of IFN-γ, IL-12p40, and TNF-α of IL-27R^-/-^ mice (n = 4) on day 7 after infection. Data are presented as the mean ± SEM. The results presented are representative of 2 separate experiments.

Having demonstrating that IL-27 is crucial for dampening trypanosomiasis-associated CD4^+^ T cell activation, needed for prolonged survival, we next addressed the mechanism of CD4^+^ T cell-mediated mortality of infected IL-27R^-/-^ mice. Because the production of IFN-γ, and the frequency and the absolute number of IFN-γ-producing cells were enhanced in infected IL-27R^-/-^ mice compared to infected wild-type mice (Fig 2 and Fig 5), and also because depletion of CD4^+^ T cells dramatically reduced the IFN-γ production (Fig 6; S2 Fig), we examined whether the early mortality of infected IL-27R^-/-^ mice was directly attributed to the overproduction of IFN-γ. IL-27R^-/-^ mice infected with *T*. *congolense* were treated with neutralizing anti-IFN-γ mAb or rat IgG as a control. Although administration of anti-IFN-γ mAb led to doubled parasitemia in infected IL-27R^-/-^ mice at the peak on day 7 after infection (P<0.05), the infected IL-27R^-/-^ mice treated with anti-IFN-γ mAb efficiently controlled the first wave of parasitemia as infected control mice did (Fig 7A). Importantly, administration of anti-IFN-γ mAb significantly enhanced the survival of infected IL-27R^-/-^ mice (p<0.01; Fig 7A), demonstrating that high levels of IFN-γ accelerated the mortality of IL-27R^-/-^ mice infected with African trypanosomes.

We next assessed the effects of IFN-γ neutralization on weight loss and liver pathology of IL-27R^-/-^ mice infected with *T*. *congolense*. Infected IL-27R^-/-^ mice treated with anti IFN-γ mAb had significantly less weight loss than infected IL-27R^-/-^ mice treated with rat-IgG on the late stage of infection (p<0.01, S3A Fig). Importantly, infected IL-27R^-/-^ mice treated with anti-IFN-γ did not exhibit areas with loss of hepatocyte cellular architecture in the liver whereas these pathological changes were observed in the liver of infected IL-27R^-/-^ mice treated with rat IgG (S3B Fig). Moreover, neutralization of IFN-γ significantly reduced the serum activities of ALT in infected IL-27R^-/-^ mice (p<0.01, Fig 7B). These data suggested that IFN-γ played a critical role in the development of liver pathology in IL-27R^-/-^ mice infected with African trypanosomes.

We finally examined cytokine responses of infected IL-27R^-/-^ mice treated with anti-IFN-γ mAb. IFN-γ was almost undetectable in the plasma of IL-27R^-/-^ mice treated with anti-IFN-γ, suggesting the neutralization was successful (p<0.01, Fig 7C). Plasma levels of IL-12p40 and TNF-α were dramatically reduced in infected IL-27R^-/-^ mice treated with anti-IFN-γ mAb, compared to infected IL-27R^-/-^ mice treated with rat IgG (p<0.01, Fig 7C). Neutralization of IFN-γ also significantly reduced the production of IL-12p40 and TNF-α by cultured spleen cells (p<0.01, or <0.05, S3C Fig). Thus, the results indicated that IFN-γ was critically involved in the enhanced inflammatory responses in IL-27R^-/-^ mice infected with African trypanosomes.

### 8. Essential role of IL-27 signaling in preventing lethal effect of CD4^+^ T cells in mice infected with *T*. *brucei*

**Fig 8:**
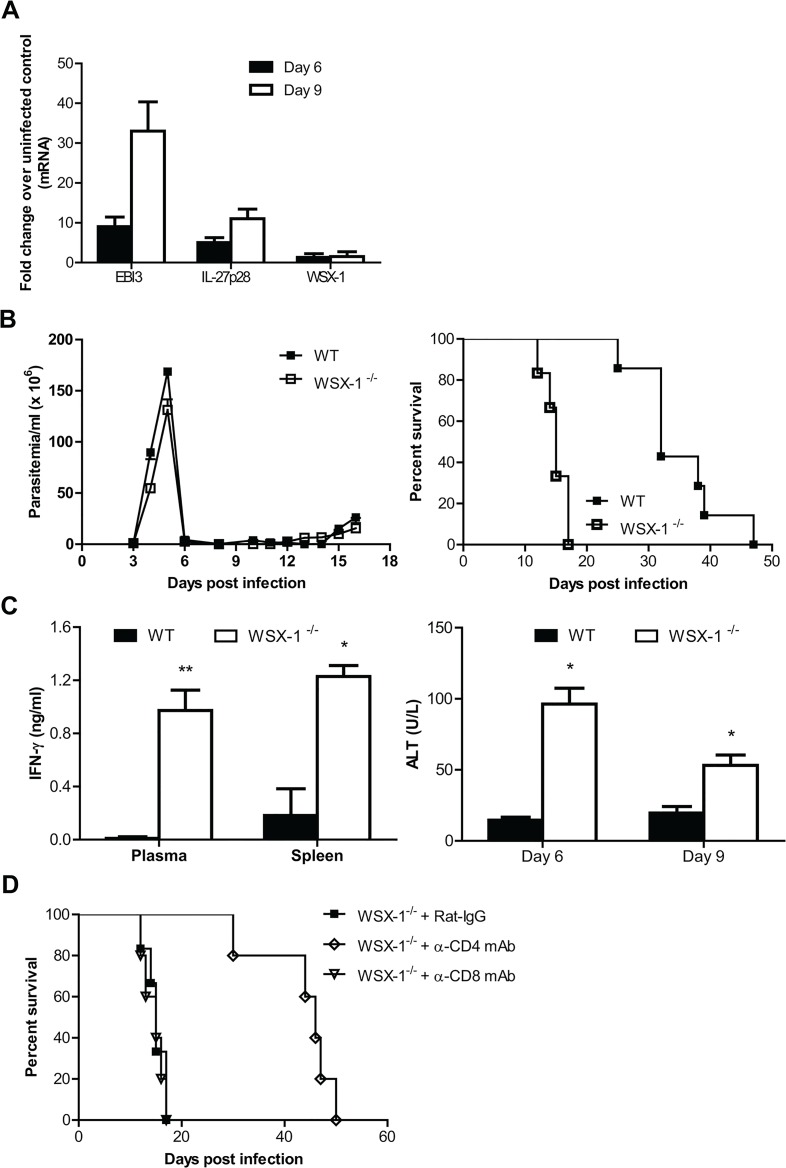
IL-27 signaling plays a crucial role in dampening Th1 mediated immune responses, allowing prolonged survival of mice infected with *T*. *brucei*. (A) mRNA expression levels of IL-27p28, EBI3 and WSX-1 in the liver of wild-type mice infected with *T*. *brucei* on day 6 and 9 versus day 0 (uninfected). (B) Parasitemia and survival of IL-27R^-/-^ (WSX-1^-/-^) and wild-type mice (n = 6–7) infected with *T*. *brucei*. (C) Production of IFN-γ detected on day 6 in the plasma and supernatant fluids of cultured spleen cells and serum activities of ALT examined on day 6 and 9 in IL-27R^-/-^ and wild-type mice after infection with *T*. *brucei*. (D) Survival of IL-27R^-/-^ mice (n = 5–6) infected with *T*. *brucei*, following administration of 0.5 mg rat anti-mouse CD4 mAb, rat anti-mouse CD8 mAb, or rat IgG on day 0, 2, 4, and 6 after infection, respectively. Data are presented as the mean ± SEM. The results presented are representative of 2–3 separate experiments.

We finally characterized the role of IL-27 signaling in regulation of immune responses during *T*. *brucei* infection. In contrast to *T*. *congolense*, *T*. *brucei* species have the ability to penetrate the walls of capillaries, invade interstitial tissues, including the brain tissues, thus serving as a model of human African trypanosomiasis [48,49]. *T*. *brucei* infection also upregulated the mRNA expressions of IL-27p28 and EBI3, but not IL-27R^-/-^ in the liver of mice (Fig 8A). IL-27R^-/-^ mice infected with *T*. *brucei* efficiently controlled the first wave of parasitemia as infected wild-type did, but survived significantly shorter than infected wild-type mice (15 days vs. 32 days, p<0.01, Fig 8B), demonstrating an essential role of IL-27 signaling in prevention of the early mortality of mice infected with *T*. *brucei*. IL-27R^-/-^ mice infected with *T*. *brucei* also showed enhanced IFN-γ production in plasma and supernatant fluids of spleen cultures, as well as enhanced serum activities of ALT, compared to infected wild-type mice (p<0.01 or <0.05, Fig 8C). Importantly, depletion of CD4^+^, but not CD8^+^, T cells enhanced the survival of IL-27R^-/-^ mice infected with *T*. *brucei* by 3 folds (p<0.01, Fig 8D). Thus, IL-27 signaling is also required for survival of mice via preventing excessive Th1 immune responses during *T*. *brucei* infection.

## Discussion

Successful clearance of African trypanosomes in the bloodstream requires induction of inflammatory immune responses; however, failure to control this inflammation leads to immune-mediated pathology [4,50]. IL-10 signaling has been previously suggested to be involved in maintaining this immunological balance in African trypanosomiasis [11,20]. In the current study, we have identified IL-27 signaling as a novel pathway to maintain this immunological balance in African trypanosomiasis. Our data are the first to demonstrate the essential role of IL-27 signaling in regulating immune responses to extracellular protozoan infections. More importantly, we provided direct evidence, that infection-associated IL-27 signaling served to extend the survival of the infected host by dampening CD4^+^ T cell activation and their secretion of IFN-γ.

Indeed, the early mortality of infected mice lacking IL-27 signaling (IL-27R^-/-^mice) was correlated with exaggerated inflammatory responses and liver immunopathology. The disease similarity of infected mice lacking IL-27 and IL-10 signaling raised the possibility that regulatory function of IL-27 is mediated via the induction of IL-10 secretion, as IL-27 has the capability of promoting CD4^+^ T cells to secret IL-10 [45–47]. However, the fact that blocking IL-10R further shortened the survival of infected IL-27R^-/-^ mice and the fact that infected mice lacking IL-10 signaling and infected mice lacking IL-27 signaling had distinct survival suggested that IL-27 functions through a mechanism independent of IL-10. In addition, compared to infected wild-type mice, infected IL-27R^-/-^ mice produced similar or even higher amounts of IL-10, depending on the time points examined. Furthermore, the enhanced survival of infected IL-27R^-/-^ mice following depletion of CD4^+^ T cells was correlated with dramatically reduced secretion of IL-10. These data suggested that a defect of IL-10 signaling is unlikely to contribute to the early mortality of IL-27R^-/-^ mice. Thus, we suggest that IL-27 suppresses the liver pathology and prevents the early mortality of mice infected with African trypanosomes through IL-10-independent mechanisms, possibly by direct modulation of T cell function.

It has been previously demonstrated that IL-10 inhibits accumulation and activation of M1-type myeloid cells, in particular, TIP-DCs (CD11b^+^Ly6C^+^CD11c^+^TNF and iNOS producing DCs) in the liver during infection with African trypanosomes [22,26,27]. Accordingly, African trypanosomes-infected CCR2 deficient mice and MIF (macrophage migrating inhibitory factor) deficient mice exhibited significantly reduced accumulation of TIP-DCs, which was correlated with remarked diminished liver pathology, and significantly prolonged survival [26,44]. Thus, IL-10 signaling suppresses liver pathology, mainly through downregulation of M1-type myeloid cells [3,50]. In contrast, IL-27R^-/-^ mice infected with African trypanosomes displayed more activation of T cells, in particular, CD4^+^ T cells. Moreover, depletion of CD4^+^ T cells prevented liver pathology and early mortality of infected IL-27R^-/-^ mice. Obviously, IL-27 signaling functions through limiting activation of CD4^+^ T cells in African trypanosomiasis. Thus, although both IL-10 signaling and IL-27 signaling are crucial for limiting the inflammatory complications associated to African trypanosome in particular in preventing liver pathology, the two signal pathways involve distinct mechanisms.

Dampening accumulation of highly activated CD4^+^ T cells by IL-27 signaling has also been recently observed in infection with other microorganisms, particularly intracellular protozoan and bacterial pathogens [38,40–42,51]. Our data demonstrate that the same mechanism exists during infections with extracellular protozoan parasites such as African trypanosomes. However, the precise mechanism of CD4^+^ T cell-mediated early mortality in previous models was not fully elucidated [38,42]. One of the most important properties of CD4^+^ T cells is that they secret a large amount of IFN-γ upon activation. IFN-γ is required to eliminate intracellular parasites, but also has potential to induce immunopathology [52,53]. Indeed, early mortality of IL-27R^-/-^ mice infected with *Toxoplasma gondii*, or *Plasmodium berghei* is associated with significantly enhanced production of IFN-γ [38,42], suggesting that IFN-γ might be a critical molecule for CD4^+^ T cell-mediated mortality in the absence of IL-27 signaling. Surprisingly, neutralization of IFN-γ did not prolong the survival, and had no effect on the liver pathology of IL-27R^-/-^ mice infected with *T*. *gondii* or *P*. *berghei* at all [38,54]. Thus, although CD4^+^ T cell-mediated mortality coincides with significantly elevated secretion of IFN-γ, it still remains inconclusive whether IFN-γ is the direct mediator of CD4^+^ T cell-dependent mortality in these infections. In contrast, neutralization of IFN-γ significantly enhanced the survival IL-27R^-/-^ mice infected with African trypanosomes accompanied by a major amelioration of liver pathology, providing direct evidence that IFN-γ directly mediated the mortality of infected IL-27R^-/-^ mice. In addition, enhanced survival of infected IL-27R^-/-^ mice depleted of CD4^+^ T cells was correlated with a dramatically reduced production of IFN-γ. Obviously, either removing of CD4^+^ T cells or neutralization of IFN-γ got rid of the lethal effect of IFN-γ, leading to the prolonged survival of infected IL-27R^-/-^ mice. Thus, another important finding of this study is that, in the absence of IL-27 signaling, CD4^+^ T cells mediated mortality directly through their secretion of IFN-γ, at least, during infection with extracellular protozoan parasites African trypanosomes.

It is important to point out that our results in no way exclude the protective role of CD4^+^ T cells and IFN-γ during infection with the parasites. Indeed, early studies have shown that there was a correlation between high IFN-γ levels in serum, low parasitemia, and host resistance during infection with African trypanosomes [18]. Subsequent studies demonstrated that VSG-specific CD4^+^ T cells mediated protection via secretion of IFN-γ [13,55]; and splenic DCs were the primary cells responsible for activating naïve VSG-specific CD4^+^ T cell responses [16,17]. The protective role of CD4^+^ T cells and IFN-γ in African trypanosomiasis has been recently confirmed by independent groups [14,15,19]. In support of previous findings, we showed that either depletion of CD4^+^ T cells or neutralization of IFN-γ resulted in a significantly elevated peak parasitemia level in IL-27R^-/-^ mice infected with *T*. *congolense*, confirming the protective role of CD4^+^ T cells and IFN-γ during the infection. It is likely that IFN-γ promotes M1-type myeloid cells to produce IL-12, TNF-α and iNOS, which has been shown to be critically involved in lysis or damage of African trypanosomes [15,21,23,25,56]. On the other hand, excessive production of IL-12, TNF-α and iNOS driven by IFN-γ could also mediate immunopathology of mice infected with African trypanosomes [22,24,26,27,57]. Further, IL-12 and TNF-α could stimulate T cells to produce more IFN-γ [4,21]. Thus, IL-10 is required to down-regulate the production of IL-12, TNF-α and iNOS possibly by direct modulation of M1-type myeloid cells [11,22,26,27]. In the present study, we identified IL-27 signaling as a novel pathway to down-regulate the secretion of IFN-γ by direct modulation of CD4^+^ T cells. Obviously, in the absence of IL-27 signaling, excessive secretions of IFN-γ by CD4^+^ T cells also mediate liver pathology and mortality, although IL-10 signaling still fully functions and the infected mice produce even more IL-10, in African trypanosomiasis. Thus, both IL-10 signaling and IL-27 signaling are required for survival of mice infected with the parasites via preventing aberrant inflammatory responses, although they function in a distinct manner in African trypanosomiasis.

In conclusion, we have described an essential role for IL-27 signaling in preventing early mortality of mice infected with African trypanosomes through dampening IFN-γ secretion by CD4^+^ T cells, thus identifying, in addition to previously described IL-10 signaling, a novel pathway for maintenance of immunological balance during infection with extracellular protozoan parasites African trypanosomes. These data contribute significantly to our understanding of both immunopathogenesis of African trypanosomiasis and mechanisms underlying IL-27 immunoregulation during infection with extracellular protozoan and bacterial pathogens.

## Materials and Methods

### Ethics statement

This study was performed in strict accordance with the recommendations in the Guide for the Care and Use of Laboratory Animals of the National Institutes of Health. The animal protocols involving mice were approved by the University of Maryland Institutional Animal Care and Use Committee (IACUC) under protocol R-12-60.

### Mice and parasites

Eight- to teen-week-old C57BL/6NCrJ (C57BL/6) mice and five- to six-week-old outbred Swiss white mice (CD1) were purchased from the National Cancer Institute (Frederick, MD). B6N.129P2-Il27ra^tm1Mak^ (IL-27R^-/-^, or WSX-1^-/-^) mice were purchased from the Jackson Laboratory and bred in-house. All animal experiments were performed in accordance with the guidelines of the Institutional Animal Care and Use Committee and Institutional Bio-safety Committee of the University of Maryland, College Park.

*T*. *congolense*, Trans Mara strain, variant antigenic type (VAT) TC13 was used in this study. The origin of this parasite strain has been previously described [58]. *T*. *brucei* AnTat1.1E was obtained from the Institute of Tropical Medicine (Antwerp, Belgium). Frozen stabilates of parasites were used for infecting CD1 mice immunosuppressed with cyclophosphamide, and passages were made every third day as described previously [58]. The parasites were purified from the blood of infected CD1 mice by DEAE-cellulose chromatography [59] and used for infecting mice.

### Antibodies

Purified rat anti-mouse IL-10 receptor (IL-10R) mAb (Clone 1B1.3a), purified rat anti-mouse CD4 mAb (Clone GK1.5), purified rat anti-mouse CD8 (Clone 53–6.72), and purified rat anti-mouse IFN-γ mAb (Clone XMG1.2) were purchased from BioXCell (West Lebanon, NH). Purified anti-mouse CD16/CD32 (FcγIII/IIR, Clone 2.4G2) were purchased from BD Biosciences. APC-Cy7 anti-mouse CD3e (145-2C11), PE-anti-mouse IFN-γ (XMG1.2), PE-Cy7-anti-mouse CD4 (GK1.5), PE-Cy7-anti-mouse CD4 (RM 4–4), FITC-anti-mouse CD8 (53–6.72), FITC-anti-mouse CD8 (YTS156.7.7), APC-anti-mouse CD44 (IM7), PE-anti-mouse CD62L (MEL-14), and matching controls were purchased from eBioscience or Biolegend.

### Infections, treatment of mice with mAbs, estimation of parasitemia and survival time of mice

Mice were infected *i*.*p*. with 10^3^ *T*. *congolense* TC13 [11] or 5×10^3^ *T*. *brucei* AnTat1.1E [44]. Some groups of infected mice were injected *i*.*p*. with rat anti-mouse IL-10R mAb (1B1.3a; 0.4 mg on day 0, 2, 4, and 6 after infection, respectively), anti-mouse CD4 mAb (GK1.5; 0.5 mg on day 0, 2, 4, and 6 after infection, respectively), anti-mouse CD8 mAb (53–6.72; 0.5 mg on day 0, 2, 4, and 6 after infection, respectively), anti-mouse IFN-γ mAb (XMG1.2; 0.4 mg on day 0, 2, 4, 6, 8, 10, 12, and 14 after infection, respectively), or rat IgG (as a control). Parasitemia was counted at ×40 magnification by phase-contrast microscopy. The survival time was defined as the number of days after infection that the infected mice remained alive.

### Detection of IL-27/WSX-1 mRNA levels

For analysis of mRNA expression, total RNA was extracted from the homogenates of the liver of uninfected wild-type C57BL/6 mice or mice infected with *T*. *congolense* or *T*. *brucei*, following the manufacturer’s recommendation (Life Technologies). IL-27p28, EBI3, and WSX-1 mRNA levels were quantified by real-time quantitative RT-PCR. The cDNA expression for each sample was standardized using the house keeping gene β-actin. Cycling conditions were as follows: initialization 2 min at 50°C and 10 min at 95°C, followed by 40 cycles of 15 s at 95°C and 1 min at 60°C. Primer pair used were: IL-27p28: 5’-CTGGTACAAGCTGGTTCCTG-3’, 5’-CTCCAGGGAGTGAAGGAGCT-3; EBI3: 5’-CAGAGTGCAATGCCATGCTTCTC-3’, 5’-CTGTGAGGTCCTGAGCTGAC-3’; WSX-1: 5’-CAAGAAGAGGTCCCGTGCTG-3’, 5’-TTGAGCCCAGTCCACCACAT-3’.

### Splenocyte or liver leukocyte cultures for measurement of cytokine synthesis

Splenocytes were collected from mice. Cells were cultured at a concentration of 5 × 10^6^ cells/ml (200 μl/well) in 96-well tissue culture plates in a humidified incubator containing 5% CO_2_. The culture supernatant fluids were collected after 48 h and centrifuged at 1,500g for 10 min, and the supernatant fluids were stored for cytokine assays at -20°C until used.

Liver leukocytes were isolated as described previously [60]. Briefly, the liver was perfused with PBS until it became pale. Thereafter, the gallbladder was removed and the liver excised carefully from the abdomen. The liver was minced into small pieces with surgical scissors and forced gently through a 70 um cell strainer using a sterile syringe plunger. The preparation obtained was suspended in 50 ml RPMI-1640 medium containing 10% FCS. The cell suspension was centrifuged at 30g with the off-brake setting for 10 min at 4°C. The obtained supernatant was centrifuged at 300g with the high-brake setting for 10 min at 4°C. The pellet was resuspended in 10 ml 37.5% Percoll in HBSS containing 100 U/ml heparin and then centrifuged at 850g with the off-brake setting for 30 min at 23°C. This new pellet was resuspended in 2 ml ACK buffer (erythrocyte lysing buffer), and incubated at room temperature for 5 min, then supplemented with 8 ml RPMI-1640 medium containing 10% FCS, followed by centrifugation at 300g with the high-brake setting for 10 min at 8°C. Cells were collected and cultured at a concentration of 5 × 10^6^ cells/ml (200 μl/well) in 96-well tissue culture plates in a humidified incubator containing 5% CO_2_. The culture supernatant fluids were collected after 48 h and centrifuged at 1,500g for 10 min, and the supernatant fluids were stored for cytokine assays at -20°C until used.

### Cytokine assays

Recombinant murine cytokines and Abs to these cytokines for use in ELISA were purchased from BD Biosciences or R&D Systems. The levels of cytokines in culture supernatant fluids or plasma were determined by routine sandwich ELISA using Immuno-4 plates (Dynax Technologies), according to the manufacturer’s protocols.

### Flow cytometry

To assess the activation of T cells, intrahepatic leukocytes were isolated as described above. The cells were incubated (15 min, 4°C) with purified anti-mouse CD16/CD32 ([FcγIII/II Receptor], clone: 2.4G2) to block nonspecific binding of Abs to FcRs, washed with staining buffer (eBioscience), resuspended in staining buffer, and stained with mAbs specific for various cell surface markers, or the relevant isotype-matched control Abs. For intracellular IFN-γ staining, spleen cells or intrahepatic leukocytes were diluted to 5 × 10^6^ cells/ml and cultured (200 μl/well) in a 96-well plate in the presence of 1x Cell Stimulation Cocktail (containing PMA, ionomycin, and protein transport inhibitors, eBioscience) for 12 h. The cells were then harvested and washed twice in staining buffer. The cells were incubated (15 min, 4°C) with purified anti-mouse CD16/CD32, washed with staining buffer, followed by staining with mAbs specific for cell surface markers. The cells were fixed and permeabilized using Intracellular Fixation & Permeabilization Buffer Set (eBiosciences). Intracellular staining was then performed using mAbs specific for IFN-γ. Samples were resuspended in staining buffer, tested by FACSAria II, and analyzed using FlowJo software.

### Aminotransferase determination and histopathological examination

Liver alanine transaminase (ALT) activities were determined using EnzyChrom Alanine Transaminase Assay Kit (BioAssay Systems) according to the manufacturer’s instructions. For histopathological examination, the liver was taken from mice on day 10 after infection and fixed with 10% formalin in PBS. Sections were stained with Hematoxylin and Eosin.

### Statistical analysis

Data are represented as the mean ± SEM. Significance of differences was determined by ANOVA or a log-rank test for curve comparison using the GraphPad Prism 5.0 software. Values of p≤0.05 are considered statistically significant.

## Supporting Information

S1 Fig Efficacy of depletion of CD4^+^ and CD8^+^ T cells by antibodies in the spleens and livers of mice infected with *T*. *congolense*. Representative flow cytometry histograms showing the depletion of CD4^+^ and CD8^+^ T cells in the spleens (A) and livers (B) of IL-27R^-/-^ (WSX-1^-/-^) mice on day 7, 10, and 14 after infection. The infected mice were administrated with 0.5 mg rat anti-mouse CD4 mAb, rat anti-mouse CD8 mAb, or rat IgG on day 0, 2, 4, and 6 after infection, respectively. The results presented are representative of 2 separate experiments.
(EPS)

S2 Fig Depletion of CD4^+^ T cells ameliorates the weight loss, prevents the development of liver pathology, reduces the production of IFN-γ by spleen cells, and abolishes the secretion of IL-10 by spleen cells in IL-27R^-/-^ (WSX-1^-/-^) mice infected with *T*. *congolense*. IL-27R^-/-^ mice were infected with 10^3^ *T*. *congolense* TC13 and treated with 0.5 mg rat anti-mouse CD4 mAb, rat anti-mouse CD8 mAb, or rat IgG on day 0, 2, 4, and 6 after infection, respectively. (A) The weight loss of infected mice (n = 4–5) was determined at indicated days after infection. (B) Hematoxylin and eosin staining was performed on the liver sections at day 10 after infection to detect pathological changes (original magnification ×20). (C) Production of IFN-γ and IL-10 in supernatant fluids of cultured spleen cells collected from mice (n = 4) at day 7 after infection. Data are presented as the mean ± SEM. The results presented are representative of 2 separate experiments.
(EPS)

S3 Fig Neutralization of IFN-γ ameliorates the weight loss, prevents the development of liver pathology, and reduces the production of IL-12p40 and TNF-α by spleen cells in IL-27R^-/-^ (WSX-1^-/-^) mice infected with *T*. *congolense*. IL-27R^-/-^ mice were infected with 10^3^ *T*. *congolense* TC13 and treated with 0.4 mg rat anti-mouse IFN-γ mAb or rat IgG on day 0, 2, 4, 6, 8, 10, 12, and 14 after infection, respectively. (A) The weight loss of infected mice (n = 5) was determined at indicated days after infection. (B) Hematoxylin and eosin staining was performed on the liver sections at day 10 after infection to detect pathological changes (original magnification ×20). (C) Production of IL-12p40 and TNF-α in supernatant fluids of cultured spleen cells collected from mice (n = 4) at day 7 after infection. Data are presented as the mean ± SEM. The results presented are representative of 2 separate experiments.
(EPS)
